# Efficient Recovery of Phosphate from Water Media by Iron-Magnesium Functionalized Lignite: Adsorption Evaluation, Mechanism Revelation and Potential Application Exploration

**DOI:** 10.3390/molecules29061252

**Published:** 2024-03-12

**Authors:** Wenbo An, Qiqi Wang, He Chen, Yifan Liu, Xuechun Hu, Junzhen Di

**Affiliations:** 1 School of Civil Engineering, Liaoning Technical University, Fuxin 123000, China; 2 School of Mining Engineering, China University of Mining and Technology, Xuzhou 221000, China; 3 School of Mechanics and Engineering, Liaoning Technical University, Fuxin 123000, China

**Keywords:** lignite, iron–magnesium functionalized, phosphate, adsorption, slow-release phosphate fertilizer

## Abstract

Selective phosphorus removal from aquatic media has become an ideal strategy to mitigate eutrophication and meet increasingly stringent discharge requirements. To achieve phosphorus control and resource utilization of low-calorific-value lignite, iron and magnesium salts were used to functionalize lignite, and iron–magnesium functionalized lignite (called IM@BC) was prepared for phosphate recovery from water media. The adsorption properties of IM@BC were systematically evaluated, especially the influence of ambient pH and co-existing ions. The kinetic, isothermal, and thermodynamic adsorption behaviors of IM@BC were analyzed. The adsorption mechanism was revealed by microscopic characterization. The potential application of phosphate-containing IM@BC (P-IM@BC) was explored. The results show that IM@BC has a strong phosphate adsorption capacity, and the maximum adsorption capacity is 226.22 mgP/g at pH = 3. Co-existing CO_3_^2−^ inhibits phosphate adsorption, while coexisting Ca^2+^ and Mg^2+^ enhance the effect. At the initial adsorption stage, the amount of phosphate adsorbed by IM@BC continues to increase, and the adsorption equilibrium state is gradually reached after 24 h. The adsorption process conforms to the pseudo-second-order kinetic model (PSO) and Langmuir isothermal adsorption model, and the adsorption process is mainly chemical adsorption. The phosphate absorption capacity is positively correlated with temperature (283.15 K~313.15 K), and the adsorption process is spontaneous, endothermic, and entropy-increasing. Its adsorption mechanism includes electrostatic attraction, ion exchange, surface precipitation, and coordination exchange. IM@BC can efficiently recover phosphate from actual phosphorus-containing wastewater with a recovery efficiency of up to 90%. P-IM@BC slowly releases phosphate from pH 3 to 11. Plant growth experiments showed that P-IM@BC could be used as a slow-release fertilizer to promote the root growth of cowpeas. The novelty of this work lies in the development of a highly efficient phosphate recovery adsorbent, which provides a feasible method of phosphorus control in water media and resource utilization of lignite.

## 1. Introduction

“Water eutrophication caused by the reduction of phosphorus resources and the loss of phosphate” are two closely related problems that have been troubling human production activities [1]. Phosphate rock is a non-renewable resource, and the exploitation of phosphorus resources is accompanied by resource shortage and environmental pollution [2]. Concomitantly with the shortage of phosphorus resources, a large amount of phosphorus is released into the environment globally every year [3]. Non-point-source phosphorus emissions from agriculture in China account for 67.20% of pollution emissions; similarly, phosphorus fertilizers account for 71% of the total phosphorus load in Italy, and agricultural resources are also a major contributor to the eutrophication of lakes and rivers in the United States [4]. Phosphorus accumulation leads to eutrophication of water bodies, and 29.1% of China’s 110 lakes are reported to be eutrophic. However, many countries and regions have developed corresponding policies and regulations to limit phosphorus emissions [5]. Therefore, the efficient removal and recovery of phosphate from water media are key measures to solve the above two problems.

In the past few decades, the adsorption method has been considered one of the most effective ways to remove phosphates from water media. However, traditional adsorbents (such as activated carbon, attapulgite, zeolite, adsorption resin, etc.) have the disadvantages of weak adsorption capacity and low affinity for phosphate [6]. To address this challenge, biochar (agricultural waste such as corn stalks and coconut husks, and natural minerals such as fly ash and coal) is a promising adsorbent for improving adsorption capacity [7,8]. Among many adsorbents, lignite stands out because of the rich functional groups on its surface. Polat et al. reported that the adsorption capacity per unit surface area of lignite is at least four times that of activated carbon [9]. However, the surface of unprocessed lignite is negatively charged, and its adsorption capacity for phosphate is limited. Samaraweera et al. also confirmed that negatively charged adsorbents can lead to poor phosphate adsorption [10]. Therefore, the functionalization of lignite to make its surface positively charged can improve its phosphate adsorption capacity.

Metal-based adsorbents have received extensive attention because of their excellent adsorption capacity and high phosphate affinity. Metal-based adsorbents mainly rely on metal ions (Ca, Mg, Al, Fe, La, etc.) to increase the number of adsorption sites and adsorption affinity on the surface of the adsorbent [11]. At the same time, these metal elements are also necessary for the growth of animals and plants and do not easily cause secondary pollution to the environment [1]. Koh et al. synthesized lanthanum carbonate nanomaterials with a phosphate adsorption capacity of 312.05 mg/g within 5 h [12]. Ahmed and Lo found that a magnetic La(OH)_3_ material reduced phosphate levels in river water from 0.089 mg/L to 0.005 mg/L [13]. Samaraweera et al. confirmed that Ca^2+^-modified lignite has better adsorption capacity and selectivity for phosphate in wastewater than unprocessed lignite [10]. Therefore, metal-based adsorbents show amazing potential in the field of phosphorus removal. It has been reported that polymetallic composite adsorbents have a higher phosphate adsorption capacity than mono-metallic adsorbents [14], which can be attributed to the synergistic effects of electrostatic attraction, surface precipitation, ligand exchange, and ion exchange [15]. Du et al. prepared a Zr–La bimetallic composite adsorbent. Under the same experimental conditions, the adsorption capacity of the bimetallic adsorbent is better than that of the single metal adsorbent, and the phosphate anion has a strong affinity with the bimetallic adsorbent [16]. The calcium/magnesium-modified lignite prepared by Samaraweera et al. also obtained similar results for the removal of phosphate from wastewater [10]. Sun et al. prepared an Fe–Mg co-modified rape straw biochar (Fe/MG-RSB), whose maximum adsorption capacity for chloroquine phosphate was 42.93 Mg/g (308 K), which was about two times higher than that of unprocessed rape straw biochar [17]. Tang et al. prepared an Fe–Mg layered dioxide-modified bagasse waste adsorbent (BM-LDO-BC), whose maximum theoretical adsorption capacity of ciprofloxacin in water reached 213.1 mg/g [18]. Up to now, few researchers have used bimetals of iron salt and magnesium salt for functionalization and modification treatment of lignite, and it is rare to use them to treat wastewater containing phosphate.

In this study, a promising adsorbent, that is, iron–magnesium functionalized lignite (called IM@BC), was for the first time prepared by using bimetals of iron and magnesium salts for the functionalization of lignite, and it was used for efficient recovery and utilization of phosphate in water media. The effects of environmental pH value and co-existing ions on the adsorption properties of IM@BC were systematically evaluated by batch experiments. The kinetic, isotherm, and thermodynamic adsorption behaviors and adsorption mechanisms of IM@BC were comprehensively analyzed. The potential application of IM@BC containing phosphate was explored. The novelty of this study lies in the proposed solution to the problem of water eutrophication caused by phosphate, and the reliable realization of phosphorus control and low-calorific-value lignite resource utilization.

## 2. Results and Discussion

### 2.1. Adsorption Characteristic

#### 2.1.1. The Effect of pH

**Figure 1:**
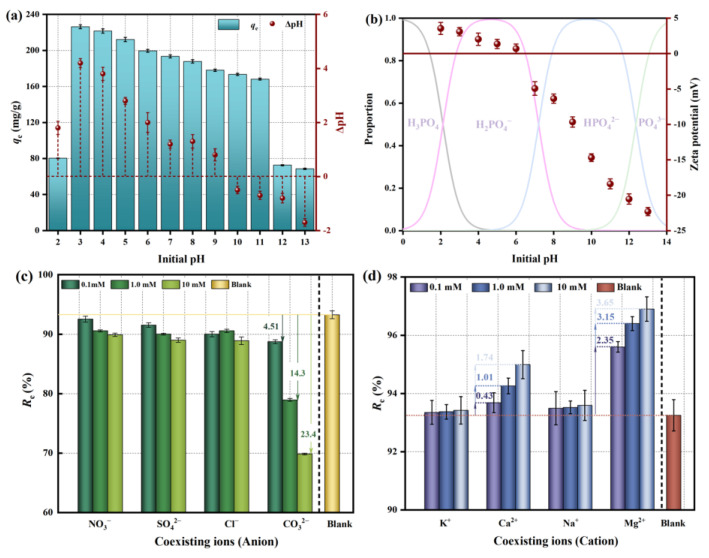
The effects of pH value and co-existing ions on phosphate adsorption by IM@BC. (**a**) pH value. (**b**) Zeta potential. (**c**) Coexisting anion ions. (**d**) Coexisting cation ions.

The removal of phosphate by adsorbent is related to the morphology of phosphate in solution and the charge on the adsorbent surface [19,20]. The morphology of phosphate in aqueous solution is affected by the pH and presents 4 morphologies: H_3_PO_4_, H_2_PO_4_^−^, HPO_4_^2−^, and PO_4_^3−^. The adsorption properties of phosphate under different pH conditions are shown in Figure 1a. When the initial pH value increased from 3 to 11, the phosphate adsorption gradually decreased. The maximum phosphate adsorption capacity of IM@BC was 226.22 mgP/g when pH was 3. The adsorption capacity of IM@BC was enhanced at the lower pH value. Acidic conditions were conducive to the driving force of electrostatic attraction, and the affinity of the active site in the surrounding environment was stronger, which was conducive to phosphate adsorption. The distribution of surface charge states for IM@BC is shown in Figure 1b. The IM@BC surface was positively charged due to the presence of Fe-C hydroxylated mineralization on the surface when the solution pH was lower than the isoelectric point (pH_ZPC_ = ~6.2), and vice versa. In other words, when pH < 6.2, positive charge dominated and electrostatic attraction promoted the absorption of phosphate, while when pH > 6.2, negative charge dominated and phosphate removal ability continued to weaken. The sludge-derived biochar prepared by Wang et al. also follows a similar rule [21]. It can also be seen from Figure 1b that the existence form of phosphate was regulated by the solution pH, and its distribution law also affected the interaction between phosphates and IM@BC adsorption sites. In the range of pH 2.0~13.0, phosphates exist in the form of H_2_PO_4_^−^ and HPO_4_^2−^, and H_2_PO_4_^−^ is more easily adsorbed than HPO_4_^2−^ due to its lower adsorption-free energy. This was also one of the reasons why the phosphate absorption capacity decreased with the increase in pH. It is worth noting that the phosphate absorption capacity is very low when pH < 2, which may be because phosphate exists in the form of neutral phosphate at this time, so the electrostatic attraction between phosphate and IM@BC is greatly weakened. When pH > 12, the phosphate absorption capacity is also very low, which may be because high pH can consume iron and magnesium ions, reducing the chance of forming a precipitate of metal salt and phosphate. At the same time, IM@BC had a negative charge and a reduced protonation, which repelled HPO_4_^2−^.

#### 2.1.2. The Effect of Co-Existing Ions

To better understand the complex situation of actual water media, the effects of representative co-existing anions (chloride, nitrate, sulfate, carbonate) and cations (K^+^, Na^+^, Ca^2+^, Mg^2+^) on phosphate adsorption by IM@BC were investigated. The effect of co-existing anions on phosphate adsorption by IM@BC is shown in Figure 1c. Co-existing anions compete with phosphates and usually inhibit their adsorption capacity. When chloride ions, nitrates, and sulfates were present in the solution, it was observed that they had a slight inhibition on the removal rate of phosphate (a negligible decrease of <5%), which means that IM@BC has a strong affinity for phosphate. The report of Qiu et al. also confirmed this phenomenon [22]. When the carbonate existed in the solution, the phosphate absorption capacity was inhibited, and the inhibition effect was strengthened with the increase in concentration level. The phosphate removal rates of 0.1 mM, 1 mM, and 10 mM carbonate decreased by 4.51%, 14.30%, and 23.40%, respectively. This was because a smaller carbonate ion radius (178 pm) was able to enhance the competitive adsorption of phosphate by IM@BC. Xu et al. also proved that the presence of carbonate could inhibit the phosphate absorption capacity, and the inhibition efficiency was 43.0% [23]. The effect of co-existing cations on phosphate adsorption by IM@BC is shown in Figure 1d. The presence of coexisting cations did not produce an inhibitory effect but enhanced the phosphate absorption capacity. The effect of Ca^2+^ and Mg^2+^ on phosphate adsorption capacity was obvious, and it was proportional to the increase in concentration level. This was because high concentrations of Ca^2+^ and Mg^2+^ can form hydroxyapatite (Ca_10_(PO_4_)_6_(OH)_2_) and Mg(PO_4_)_2_ precipitates with phosphate.

### 2.2. Adsorption Kinetics

**Figure 2:**
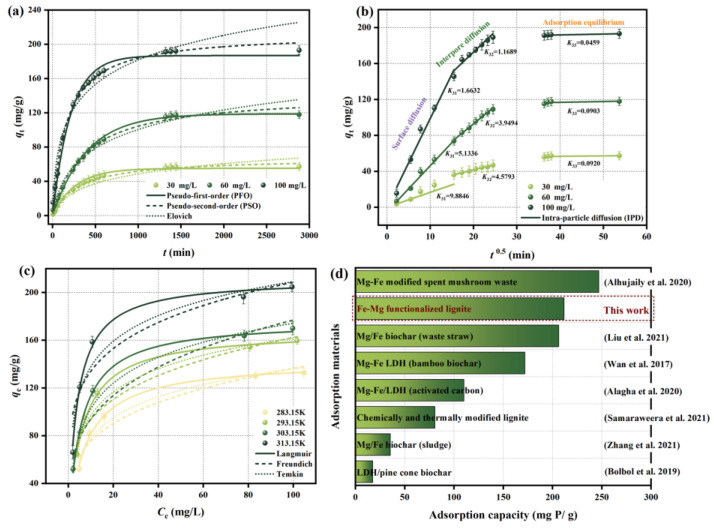
The adsorption properties and capacity of phosphate by IM@BC. (**a**) Adsorption kinetics. (**b**) Adsorption isotherm. (**c**) Adsorption thermodynamics. (**d**) IM@BC comparison of adsorption capacity with other similar adsorbents [10,24,25,26,27,28,29].

Figure 2a,b shows pseudo-first-order (PFO), pseudo-second-order (PSO), Elovich, and intra-particle diffusion (IPD) kinetics models of phosphate adsorbed by IM@BC. Their fitting parameters are shown in Table 1. At the initial adsorption stage, the amount of phosphate adsorbed by IM@BC continued to increase, and the adsorption equilibrium state was gradually reached after 24 h. The adsorption capacity was positively correlated with the initial concentration of phosphate. It can be seen from Figure 2a and Table 1 that the PSO model was more suitable for describing the phosphate adsorption process of IM@BC (*R*^2^ was the highest), indicating that the process was controlled by chemisorption and may be related to electron sharing or electron transfer on the adsorbent surface. It can be seen from Figure 2b and Table 1 that the IPD model divided the adsorption process into three stages, namely, rapid surface diffusion, intra-particle diffusion, and adsorption equilibrium. Compared to the adsorption rate *K*_3i_ and boundary layer thickness *C*_i_ of those three stages, the adsorption process was mainly dominated by the rapid surface diffusion stage. It is worth noting that the model-fitting curves of each stage did not pass through the origin, indicating that the adsorption process was a complex process controlled by multiple factors.

### 2.3. Adsorption Isotherms

Figure 2c shows Langmuir, Freundlich, and Temkin isotherm models of phosphate adsorbed by IM@BC. Their fitting parameters are shown in Table 2. In the low concentration range (~70 mg/L), the phosphate adsorption capacity of IM@BC increased sharply with the increase in initial phosphate concentration, and then the adsorption equilibrium was gradually reached. The adsorption capacity was positively correlated with temperature, and the maximum phosphate adsorption capacity was obtained at 313.15 K. It can be seen from Figure 2c and Table 2 that, compared with the Freundlich and Temkin models, the Langmuir model could better describe the phosphate adsorption process (*R*^2^ was the highest), indicating that the adsorption process conformed to the characteristics of monolayer adsorption. The surface of the adsorbent was covered by a single layer of phosphate [30], and the adsorbent surface had a strong affinity between adsorbed ions [31]. In addition, the increase in temperature could improve the movement rate of phosphate ions, and *q*_max_ gradually increased, reaching a maximum of 211.6025 mg P/g at 313.15 K. The adsorption capacities of IM@BC and other similar adsorbents (the lignite modified by different methods and the biochars functionalized with iron and magnesium salts) are compared in Figure 2d. It can be seen that the high adsorption capacity of IM@BC and its effective use as a lignite resource highlight its potential to recover phosphate from eutrophication water.

### 2.4. Adsorption Thermodynamics

In the range of 283.15~313.15 K, there was a positive correlation between the phosphate adsorption capacity of IM@BC and temperature. The thermodynamic parameters of adsorption are shown in Table 3. Δ*G* < 0 means that IM@BC has a spontaneous adsorption of phosphate, and Δ*G* decreases with increasing temperature, indicating that high temperature promotes the adsorption process. Δ*H* > 0 means that IM@BC is an endothermic reaction. We hypothesize that the endothermic process of the whole system is due to the heat absorbed by the dehydration process exceeding the heat released by the IM@BC surface ions. Δ*H* is 23.546 kJ/mol (20~40 kJ/mol), indicating that the phosphate adsorption by IM@BC is controlled by both chemical and physical action. ΔS > 0 means that the adsorption process contributes to the entropy increase in the entire system, due to the release of water molecules and Cl^−^ from the interlayer and the hydration loss of phosphate species. Other factors, such as increased randomness of the liquid–liquid interface, may also lead to an increase in overall entropy.

### 2.5. Adsorption Mechanism

#### 2.5.1. The Results of BET

**Figure 3:**
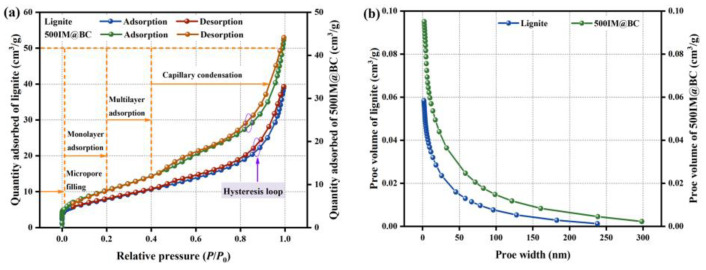

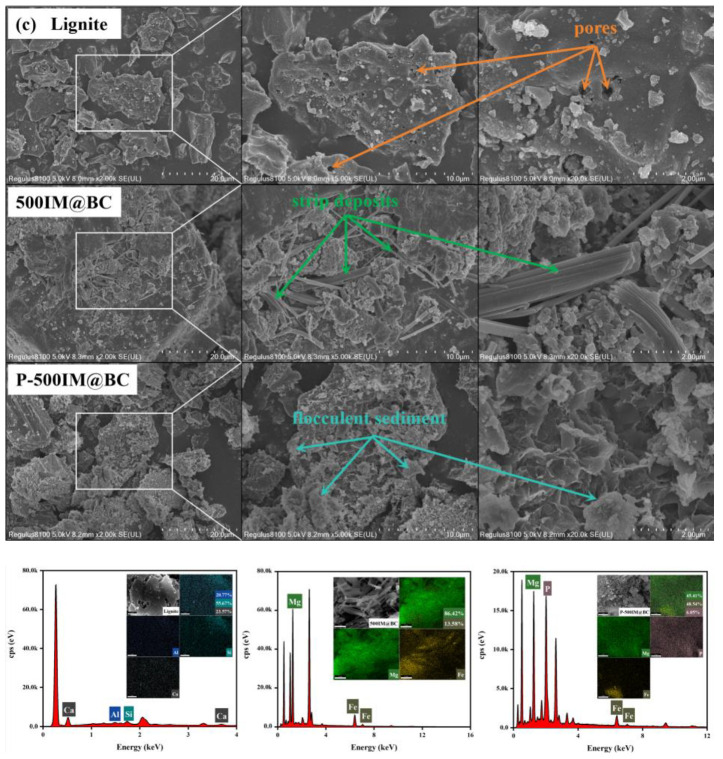
Microscopic characterization results of phosphate adsorbed by IM@BC. (**a**,**b**) BET. (**c**) FESEM-EDS.

After the iron–magnesium functionalization treatment, the specific surface area of lignite was increased from 28.552 m^2^/g to 32.052 m^2^/g, and the pore volume of lignite was increased from 1.109 cm^3^/g to 1.695 cm^3^/g, which may be due to the metal oxide loading on the IM@BC surface [32]. At the same time, although high-temperature pyrolysis will cause the collapse and blockage of the original pore structure [33], the small molecular gases released by the decomposition of organic substances in lignite will produce new pore structures. The N_2_ adsorption–desorption isotherms of lignite and IM@BC are shown in Figure 3a. These two types of adsorption isotherms showed the same trend under different relative pressures, which accorded with the typical type IV. The resulting hysteresis ring belonged to the H3 type, and the hysteresis ring started at *P*/*P*_0_ > 0.4, indicating that a mesoporous structure was formed based on capillary condensation of N_2_ in the pore [34]. The pore-size distribution curves of lignite and IM@BC are shown in Figure 3b. Their mean pore sizes (33.633 nm for lignite and 43.857 nm for IM@BC) were dominated by mesoporous (2–50 nm). The average pore size of the phosphate ion radius in aqueous solutions was 0.238 nm [35], which means that they could achieve effective phosphate adsorption through pore filling.

#### 2.5.2. The Results of FESEM-EDS

The FESEM-EDS images of lignite, IM@BC, and P-IM@BC are shown in Figure 3c. A large number of pore structures and folds appeared in the images of lignite and IM@BC, and these irregular structures were positively correlated with their porosity and specific surface area. These characteristics were the strong support for the good adsorption capacity of the adsorbent. Compared with lignite, a large number of strip-shaped deposits appeared on the surface of IM@BC, distributed in the surface and pores. These deposits may be metal oxides formed by the original metal hydroxide at high temperatures. The results of EDS mapping showed that the distribution of Fe and Mg elements was consistent with the regional layout of the bars, and it was speculated that the bar shape deposits may be Fe_3_O_4_ and MgO. The relative content of Mg element in this region was more than that of Fe, indicating that Mg element provided a greater contribution to the functionalization of lignite. In addition, compared with IM@BC, a large number of secondary flock-like crystal structures appeared after the disappearance of the bars in the adsorption saturation P-IM@BC, which may be related to the deposition of white crystalline precipitates (Fe-P, Mg-P complexes) produced by phosphate, iron and magnesium ions. In other words, surface chemical precipitation was one of the important adsorption mechanisms [36]. EDS results detected a P element, indicating that phosphate was successfully adsorbed by IM@BC.

#### 2.5.3. The Results of XRD

**Figure 4:**
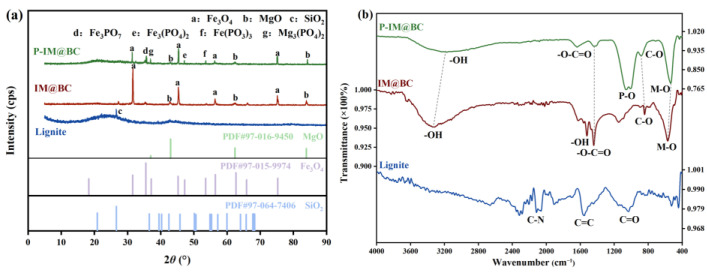
Microscopic characterization results of phosphate adsorbed by IM@BC. (**a**) XRD. (**b**) FTIR.

The XRD patterns of lignite, IM@BC, and P-IM@BC are shown in Figure 4a. The peak value of 26.3° in the lignite spectrum corresponded to the reflection of quartz, and the prominent part between 20 and 30° belonged to the characteristic structure of organic matter in lignite. Compared with lignite, after iron–magnesium functionalization, Fe_3_O_4_ and MgO reflections appeared in the IM@BC spectrum. Specifically, the diffraction peaks at 31.54°, 45.25°, 56.46°, and 75.29° corresponded to the plane reflections of (2, 2, 0), (4, 0, 0), (5, 1, 1), and (4, 2, 2) of Fe_3_O_4_ (PDF#97-015-9974), and the diffraction peaks at 42.96°, 62.39°, and 83.92° corresponded to the plane reflections of (1, 1, 1), (2, 0, 0), and (2, 2, 0) of MgO (PDF#97-016-9450). It can also be further confirmed that the strip deposits in the SEM images were metal oxides (Fe_3_O_4_ and MgO). The prominent organic characteristic peaks between 20° and 30°disappeared, which indicated that high-temperature pyrolysis would decompose organic matter in lignite and lead to changes in crystal structure, confirming the emergence of new pore structures. After phosphate adsorption, the reflection of Fe_3_PO_7_ (PDF#00-014-0147), Fe_3_(PO_4_)_2_ (PDF#00-014-0337), Fe(PO_3_)_3_ (PDF#00-013-0263), Mg_3_(PO_4_)_2_ (PDF#00-019-0767) precipitates appeared in the P-IM@BC spectrum, which proved that the flocculated crystal material in the SEM image was the deposition of Fe-P and Mg-P complexes formed by magnesium ion, iron ion and phosphate.

#### 2.5.4. The Results of FTIR

The FTIR spectra of lignite, IM@BC, and P-IM@BC are shown in Figure 4b. Compared with lignite, a new stretching vibration characteristic peak of -OH was added near 3330 cm^−1^ and 1515 cm^−1^ in the spectrum of IM@BC, indicating the increase in alcohol or phenolic functional groups after iron–magnesium functionalization. The C=O peak near 1440 cm^−1^ belonged to the antisymmetric tensile vibration of carbonate, which could be attributed to soluble CO_2_ and Fe(OH)_3_ and Mg(OH)_2_ during the material synthesis process. The stretching vibration characteristic peak of M-O appeared near 570 cm^−1^ (M was Mg or Fe), which confirmed the feasibility of iron–magnesium functional treatment of lignite. In addition, the infrared peak corresponding to the O=C-O- antisymmetric tensile vibration near 1437 cm^−1^ could be observed in the IM@BC spectrum, which could be attributed to the ion exchange reaction between the carbonate and the phosphate anion. Jiang et al. also obtained similar results, and these carbonates may come from the CO_2_ absorbed from the air during the material synthesis process [37]. A new P-O characteristic peak was added near 1056 cm^−1^, which was attributed to the v3 band vibration of HPO_4_^2−^ or H_2_PO_4_^−^, indicating that IM@BC successfully adsorbed phosphate.

#### 2.5.5. The Results of XPS

**Figure 5:**
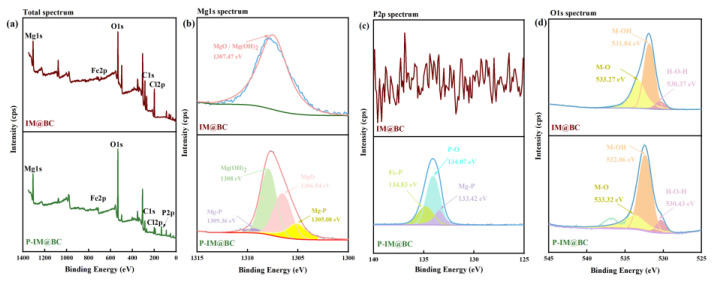
Microscopic characterization results of phosphate adsorbed by IM@BC. (**a**) Total spectrum of XPS. (**b**) Mg1s spectrum of XPS. (**c**) P2p spectrum of XPS. (**d**) O1s spectrum of XPS.

To further reveal the adsorption mechanism of phosphate by IM@BC, XPS detection of IM@BC was performed before and after the adsorption reaction (Figure 5a). The total spectrum, Mg1s, P2p, and O1s peak spectra were observed. The spectra changed greatly before and after the adsorption reaction. Figure 5b shows the total spectrum. Mg and Fe appeared in the total spectrum before the adsorption reaction, and P was also detected in the total spectrum after the adsorption reaction, which proved that IM@BC successfully adsorbed phosphate. Figure 5c shows the Mg1s spectrum. Before the reaction, there was an obvious peak at 1307.47 eV, which belonged to metal oxides, namely magnesium oxide or magnesium hydroxide. After the reaction, there were obvious peaks at 1309.36 eV, 1307.92 eV, 1306.54 eV, and 1305.08 eV, which belonged to the non-metallic peaks of Mg element (Mg-P). Figure 5d was the P2p spectrum. No P2p peak appeared before the reaction, but obvious peaks appeared at 134.83 eV, 134.07 eV, and 133.42 eV after the reaction. These peaks belonged to metal phosphorus peaks (Mg-P or Fe-P), indicating that Fe, Mg, and P elements had combined.

#### 2.5.6. The Results of 2D-COS

**Figure 6:**
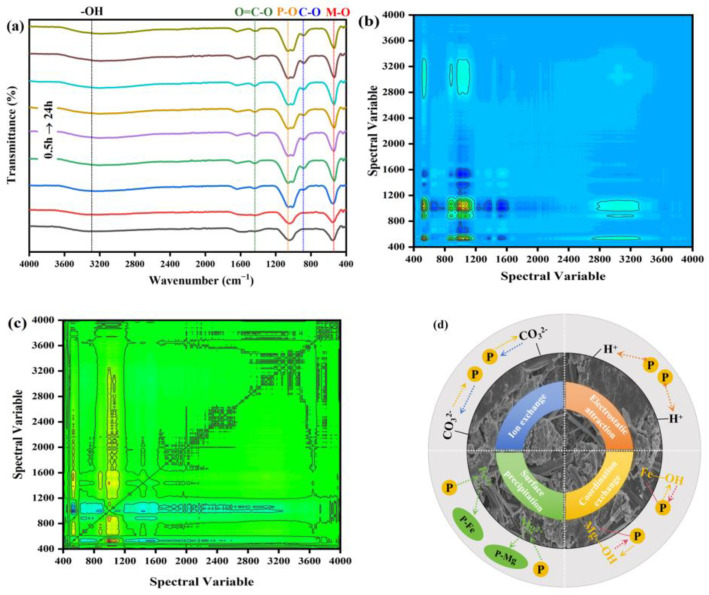
(**a**) FITR spectra of phosphate adsorbed by IM@BC at different adsorption times. (**b**,**c**) Synchronous and asynchronous spectra of 2D-COS analysis. (**d**) Schematic diagram of the adsorption mechanism.

To further explore the adsorption mechanism, FITR spectral data of phosphate adsorbed by IM@BC at different adsorption times (0.5 → 24 h) were taken as reference (Figure 6a). It can be seen from Figure 6a that the functional groups (-OH, C-O, O=C-O, P-O, M-O) were changed. The increase in -OH with time indicated that hydration occurs during the adsorption process. The increase in C-O and O=C-O was attributed to the ion exchange of carbonates in the solution. The gradual increase in P-O indicated that the adsorption amount of PO_4_^3−^ was positively correlated with the adsorption time. The reduction in M-O indicated that Fe and Mg elements participated in the adsorption process and were gradually consumed over time. Two-dimensional correlation spectroscopy (2D-COS) was used to determine the order of surface functional groups participating in the adsorption reaction [38]. The sequence of functional group changes could be evaluated by the symbols of cross-peaks in the synchronous and asynchronous spectra (Figure 6b,c). A synchronous map (Figure 6b) shows major auto-peaks appearing in the diagonal line. The positive cross-peaks of two bands showed the variations of spectra occurring under the same orientation. An asynchronous map can represent the variance of spectral intensity (Figure 6c). Cross-peaks appeared at 1150, 1437, 1525, 564, and 1056 cm^−1^ in the asynchronous map, and there were different phases between the dynamic spectral intensity changes in the two wave numbers. These peaks on a nondiagonal line were positively–negatively correlated [39,40]. The characteristics of autocorrelation and cross-correlation peaks in synchronous and asynchronous two-dimensional correlation spectra could be explained by Noda rules. In short, if a spike (v_1_, v_2_) has the same sign in both spectra (whether positive +v_e_ or negative −v_e_), then the change in v_1_ preceded the change in v_2_. Conversely, if the sign is different, then the change in v_2_ occurred first. Table 4 shows the cross-peak symbols for synchronous and asynchronous mappings and their multiplication results in IM@BC. The order of peak vibration was 1150 cm^−1^ < 1437 cm^−1^ < 1525 cm^−1^ < 564 cm^−1^ < 1056 cm^−1^; that is, the order of major functional groups involved in the phosphate adsorption process was C-O < O=C-O < -OH < M-O < P-O.

In summary, the phosphate adsorption mechanism of IM@BC is expressed in detail in Figure 6d. It mainly includes electrostatic attraction, ion exchange, surface precipitation, and coordination exchange.

### 2.6. Application Potential of Saturated Adsorbents

**Figure 7:**
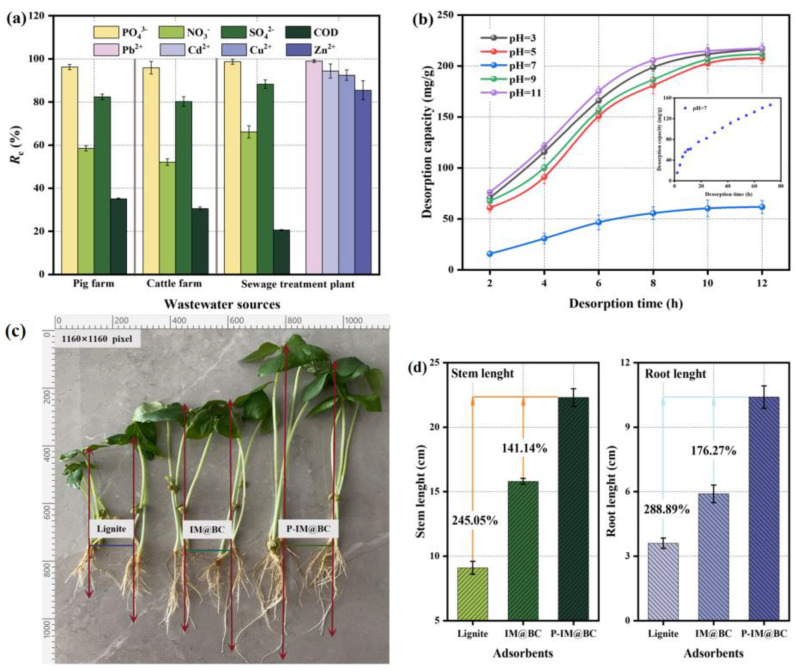
Application potential of adsorbents. (**a**) Application of IM@BC in actual wastewater. (**b**) Desorption experiment results of P-IM@BC. (**c**,**d**) Plant growth experiment results.

The adsorption capacity of IM@CB for actual phosphorus-containing wastewater from different sources (pig farm, cattle farm, sludge supernatant of sewage treatment plant) is shown in Figure 7a. In the wastewater from the three sources, the phosphate removal rate of IM@CB was still at a high level, and the removal rate was above 95%. Compared with the three, the nitrate and sulfate contained in the wastewater of pig farms and cattle farms could inhibit the adsorption of IM@BC phosphate, but the inhibition effect was weak, which was consistent with the results of the coexistence ion adsorption experiment. The relatively high removal rate of phosphate in the sludge supernatant was also due to the co-existence of heavy metal ions in the supernatant that would enhance the adsorption. At the same time, the removal rate of heavy metal ions in the supernatant was also maintained at a high level, and the removal rate of Pb^2+^, Cd^2+^, and Cu^2+^ reached more than 90%. It can be seen that IM@CB had a high adsorption capacity for actual wastewater containing phosphorus.

At different pH levels, the change rule of phosphate desorption in saturated P-IM@BC with time is shown in Figure 7b. At the initial stage, the amount of phosphate desorption in the solution generally increased and reached a stable level after 12 h. In addition, solution pH could also significantly affect the release of phosphate by saturated adsorbents. Acidic and alkaline solutions had a better desorption effect on phosphate, and neutral solutions had the least desorption. In acidic desorption solution (pH = 3), the desorption amounts of phosphate in saturated P-IM@BC were 64.26% and 55.23%, respectively. It may be because in acidic environments, desorbed PO_4_^3−^ was able to combine with H^+^ to form chemically stable H_3_PO_4_, which promoted phosphate desorption. In the alkaline desorption solution (pH = 11), the phosphate desorption amounts in the saturated P-IM@BC were 70.65% and 60.29%, respectively. This was because, in the alkaline environment, there was a competitive adsorption relationship between more OH^−^ and PO_4_^3−^ in the solution on the surface of IM@BC, which would also promote the desorption of phosphate. Therefore, phosphate release is faster in acidic and alkaline environments. It can also be observed from Figure 7b that saturated P-IM@BC has the least desorption and slowest release in the neutral solution. Therefore, the sustained-release ability of phosphate saturated with P-IM@BC over a longer period (72 h) continued to be investigated. It was found that phosphate (146.52 mg/g) was slowly released from P-IM@BC until 72 h saturation. Therefore, saturated P-IM@BC can slowly release phosphate in a neutral solution. In addition, the phosphate release in P-IM@BC is much higher than the ideal phosphorus for growing plants and producing crops (45–50 mg/kg) [41]. It is feasible to use P-IM@BC as phosphate fertilizer in agricultural production.

The growth of cowpea seedlings after 7 days of application of lignite, IM@BC, and P-IM@BC as fertilizers is shown in Figure 7c. By observing the root length and stem length of cowpea seedlings (Figure 7d), it was found that, compared with lignite and IM@BC, P-IM@BC as phosphate fertilizer significantly increased the stem length and root length of cowpea and had a significant promoting effect on the growth of seedlings. For example, the application of P-IM@BC increased stem length by 245.05% and 141.14% and root length by 288.89% and 176.27%, respectively, compared with the application of lignite and IM@BC. According to SEM-ESD results (Figure 3c), the relative content of the P element in P-IM@BC was 6.05%, which is required for plant growth. Yao et al. [41] and Zhao et al. [42] also reported that phosphorus-containing biochar could promote seed germination and seedling growth.

## 3. Materials and Methods

### 3.1. Materials and Chemicals

Lignite was taken from a coal mine in Datong City, Shanxi Province, China. It was used as raw material for adsorbent production after being washed in deionized water, dried at 105 °C, broken, and passed through 80-mesh screens. Hydrochloric acid and nitric acid were purchased from Sinopharm Group Chemical Reagent Co., Ltd. (Shanghai, China). Ammonium molybdate, potassium persulfate, and ascorbic acid were purchased from Liaoning Quanrui Reagent Co., Ltd. (Shenyang, China). Magnesium chloride hexahydrate, ferric chloride, potassium dihydrogen phosphate, potassium antimony tartrate, sulfuric acid, and sodium hydroxide were purchased from Tianjin Fuchen Chemical Reagent Co., Ltd. (Tianjin, China). All solutions were prepared with deionized water. All glassware was soaked in a 10% nitric acid solution for at least 24 h and then ultrasonically cleaned.

### 3.2. Preparation of Iron–Magnesium Functionalized Lignite (IM@BC)

The IM@BC was prepared by loading iron and magnesium salts onto the surface of lignite. First, the prepared lignite was impregnated into a mixture of 100 mL FeCl_3_ and MgCl_2_·6H_2_O for 8 h. The solution had a pH of 10 and a molar ratio of iron to magnesium of 1:2. Then, sodium hydroxide solution was added to the mixture. The impregnation solution was oscillated at 30 °C and 150 rpm for 30 min to trigger the co-precipitation reaction, resulting in Fe(OH)_3_ and Mg(OH)_2_ being loaded onto the lignite surface. The impregnation solution was left to stand for 24 h, filtered, repeatedly washed with deionized water, and dried at 80 °C for 12 h to remove the adsorbed water in the surface layer. Finally, the iron–magnesium functionalized lignite obtained in the previous stage was pyrolyzed in a Muffle furnace. Pyrolysis at 500 °C for 2 h converted Fe(OH)_3_ and Mg(OH)_2_ into Fe_2_O_3_ and MgO, and the IM@BC was obtained.

### 3.3. Batch Adsorption Experiments

The effects of pH value, co-existing ions (anions and cations), contact time, initial concentration, and system temperature on phosphate adsorption by IM@BC were investigated. The IM@BC was added into the phosphate solution at 0.2 g/L (concentration was 60 mg/L and pH was 7). The batch experiment was carried out in a 150 mL conical flask and oscillated at 30 °C and 150 rpm for 24 h until equilibrium adsorption. The simulated wastewater with different initial concentrations (30, 60, and 100 mg/L) was prepared by using a potassium dihydrogen phosphate solution. The initial pH was adjusted to 2~13 by using 0.1 M diluted hydrochloric acid or sodium hydroxide. The samples were extracted and filtered (0.45 μm) at different time intervals. The concentration of residual phosphate in the solution was determined. The removal rate (*R*_e_), adsorption capacity at time *t* (*q*_t_), and equilibrium adsorption capacity (*q*_e_) were calculated according to Equations (1), (2), and (3), respectively [43,44].

(1) $$R_{e}=\frac{C_{0}-C_{t}}{C_{0}}\times 100\%$$

(2) $$q_{t}=\frac{\left(C_{0}-C_{t}\right)V}{M}\times 100\%$$

(3) $$q_{e}=\frac{\left(C_{0}-C_{e}\right)V}{M}\times 100\%$$

where *C*_0_ is the initial mass concentration (mg/L), *C*_t_ is the mass concentration of the adsorbent in the solution at time *t* (mg/L), *C*_e_ is the mass concentration of the adsorbent in the solution at equilibrium (mg/L), *V* is the volume of the solution (L), and *M* is the mass of the adsorbent (g).

### 3.4. Data Analysis and Modelling

Four nonlinear adsorption kinetics models [45,46] (pseudo-first-order (PFO), pseudo-second-order (PSO), Elovich, and intra-particle diffusion (IPD)) were used to fit the experimental data points to obtain the most suitable description of phosphate adsorption kinetics. Three classical nonlinear adsorption isotherm models [47,48] (Langmuir, Freundlich, and Temkin) were used to fit the experimental data points to obtain the most suitable description of phosphate adsorption isotherms. The equations and parameters of these models are described in detail in Table 1. The goodness of fit and accuracy of the model were evaluated by the coefficient of determination (*R*^2^). Gibbs free energy Δ*G*, entropy change Δ*H*, and enthalpy change Δ*S* were used to describe the adsorption thermodynamic behavior of phosphate [49,50].

### 3.5. Characterization of Adsorbents

Specific surface area (*S*_BET_), total pore volume (*V*_tot_), and pore-size distribution of adsorbents were studied by using a specific surface area and porosity analyzer (BET, ASAP2020, Micromeritics, Norcross, GA, USA). The morphology and elemental information of adsorbents were studied by field emission scanning electron microscopy combined with energy dispersion spectroscopy (FESEM-EDS, Regulus 8100, Hitachi, Japan). The crystal structure of the adsorbent was studied by X-ray diffraction (XRD, D8 Advance, Bruker, Germany). The surface functional groups of adsorbents were detected by Fourier transform infrared spectroscopy (FTIR, Nicolet iS10, Thermo Scientific^TM^, Waltham, MA, USA). The elemental binding energy of adsorbents was studied by X-ray photoelectron spectroscopy (XPS, EscaLab 250Xi, Thermo Scientific^TM^, USA). The charge state on the surface of the adsorbent was detected using the Zeta potential analyzer (Zetasizer Nano ZS90, Malvern Instruments LTD, Malvern, UK).

To identify the subtle changes in FTIR spectra and the structural changes of IM@BC after phosphate adsorption, two-dimensional correlation spectroscopy (2DCOS) was used to further analyze FTIR absorption spectra. Using the adsorption time (0.5, 1, 3, 5, 6, 7, 9, 12, 24 h) as a reference, the FTIR dataset was converted into a new spectral matrix for 2DCOS analysis. Details about 2DCOS can be found elsewhere [51].

The pH value in wastewater was determined by the glass electrode method (GB/T 6920-86) [52]. Heavy metal ion (Pb, Cd, Zn, Cu) concentrations were measured through an atomic absorption spectrometer method (GB 7475-87). The wavelengths used for the analysis of the Pb, Cd, Zn, and Cu were 283.3, 228.8, 213.8, and 324.7 nm, respectively. The phosphate in wastewater was determined by the ammonium molybdate spectrophotometric method (GB 11893-89). The sulfate in wastewater was determined by barium chromate spectrophotometry (HJ/T 342-2007). The nitrate in wastewater was determined by the spectrophotometric method with phenol sulfonic acid (GB 7480-87).

### 3.6. Regeneration and Utilization Experiments

The application potential of IM@BC in wastewater containing phosphorus from different sources was evaluated by adsorption experiments. The actual wastewater was taken from a pig farm, cattle farm, and sludge supernatant of a sewage treatment plant. The wastewater was collected in 5-litre polypropylene bottles, carried at a constant temperature of 4 °C, and tested on the day of collection. The adsorption capacity of IM@BC for pH, heavy metals (Pb, Cd, Cu, and Zn), anions (phosphate, nitrate, sulfate), and organic matter (COD) in wastewater was determined by adding 0.2 g/L IM@BC under the same reaction conditions as the batch adsorption experiment.

Phosphate slow-release experiments were used to evaluate the phosphate release ability of saturated adsorbents at different pH levels. The pH value of the desorption solution was adjusted to the range of 3~11 using 0.1 M hydrochloric acid or sodium hydroxide. The saturated IM@BC containing phosphate (60 mg P/L, pH = 7) was rinsed with deionized water several times and dried in an oven at 80 °C for 24 h. The 0.2 g/L dried IM@BC was added to the desorption solution and oscillated in a shaker at 150 rpm and 25 °C for 2,4,6,8,10, and 12 h, respectively. After desorption, the samples were extracted and filtered (0.45 μm) to determine the phosphate concentration in the solution. In addition, the desorption of the saturated IM@BC was continued in a neutral aqueous solution. The desorption time was 72 h, and the other experimental conditions were consistent with the above.

Plant growth experiments were used to evaluate the potential of phosphorus-containing adsorbents as slow-release phosphate fertilizers. The dried IM@BC containing phosphate was mixed with the growing soil at a mass ratio of 1:100, as with the soil containing phosphate fertilizer. Well-grown cowpea seeds with buds longer than 0.5 cm were screened and planted in the soil. The stem lengths and root lengths of cowpea seedlings were measured and analyzed after culture at room temperature and under normal light conditions for 7 days.

## 4. Conclusions

Iron–magnesium functionalized lignite (IM@BC) was for the first time successfully prepared using bimetallic lignite loaded with iron and magnesium salts, and it can be used to recover phosphate from water media. IM@BC has a strong phosphate adsorption capacity, which is affected by the environmental pH value and co-existing ions. The adsorption process underwent three stages: rapid surface diffusion, diffusion in pores, and adsorption equilibrium. The adsorption process conforms to the pseudo-second-order kinetic model (PSO) and Langmuir isothermal adsorption model. The adsorption process is dominated by chemisorption, and the adsorption is spontaneous, endothermic, and entropy-increasing. The adsorption mechanism includes electrostatic attraction, ion exchange, chemical precipitation, and coordination exchange. IM@BC provides efficient recovery of phosphate from actual phosphorus-containing wastewater. P-IM@BC can release phosphate slowly and can be used as a slow-release fertilizer to promote the root growth of cowpeas. The novelty of this work lies in the development of a highly efficient phosphate recovery adsorbent, which provides a feasible method of phosphorus control in water media and resource utilization of lignite.

## Figures and Tables

**Table 1:** Adsorption kinetic parameters for adsorption of phosphate by IM@BC.

**Initial Concentration of Phosphate (mg/L)**	**Model**								
	**Pseudo-First-Order (PFO)**			**Pseudo-Second-Order (PSO)**			**Elovich**		
	$\ln(q_{e}-q_{t})=\ln q_{t}-K_{1}t$			$\frac{t}{q_{t}}=\frac{1}{K_{2}q_{e}^{2}}+\frac{t}{q_{e}}$			$q_{t}=\frac{1}{\beta }\ln(\alpha \beta )+\frac{1}{\beta }\ln t$		
	*K*_1_	*q*_e_	*R* ^2^	*K*_2_	*q*_e_	*R* ^2^	*α*	*β*	*R* ^2^
30	0.00376	55.1172	0.9286	4.90 × 10^−5^	67.2843	0.9347	0.2447	0.0549	0.9325
60	0.00250	118.7606	0.9910	1.97 × 10^−5^	141.6509	0.9913	0.6093	0.0292	0.9842
100	0.00472	186.7815	0.9870	2.97 × 10^−5^	212.5615	0.9948	3.1541	0.0239	0.9509
**Initial Concentration of Phosphate (mg/L)**	**Intra-Particle Diffusion (IPD)**								
	$q_{t}=k_{i}\cdot t^{0.5}+C$								
	*K*_31_	*C*_1_	*R*_1_^2^	*K*_32_	*C*_2_	*R*_2_^2^	*K*_33_	*C*_3_	*R*_3_^2^
30	1.6632	−0.0325	0.9831	1.1689	18.2824	0.9911	0.0459	54.8854	0.9483
60	5.1336	−5.1105	0.9902	3.9494	13.8898	0.9929	0.0903	113.1805	0.9974
100	9.8846	−0.0239	0.9792	4.5793	81.1255	0.9299	0.0920	188.1738	0.9916

**Table 2:** Adsorption isotherm parameters for adsorption of phosphate by IM@BC.

**Temperature (K)**	**Model**								
	**Langmuir**			**Freundlich**			**Temkin**		
	$\frac{C_{e}}{q_{e}}=\frac{1}{q_{m\mathrm{ax}}K_{L}}+\frac{C_{e}}{q_{max}}$			$\log q_{e}=\log K_{F}+\frac{1}{n}\log C_{e}$			$q_{e}=B\ln A+B\ln C_{e}$		
	*q*_max_	*K*_L_	*R* ^2^	*K*_F_	1/*n*	*R* ^2^	*A*	*B*	*R* ^2^
283.15	142.8071	0.1316	0.9940	41.7358	−0.2572	0.9358	0.0812	25.4969	0.9688
293.15	167.8054	0.1678	0.9983	53.9760	−0.2390	0.9457	0.1124	28.1210	0.9749
303.15	176.5077	0.1784	0.9948	49.8908	−0.2748	0.9583	0.0858	31.1722	0.9928
313.15	211.6025	0.2503	0.9959	85.9176	−0.1926	0.9157	0.2716	31.0343	0.9544

**Table 3:** Adsorption thermodynamic parameters for adsorption of phosphate by IM@BC.

**Pollutants**	**Δ*H*** **(kJ/mol)**	**Δ*S*** **(J/mol·K)**	**Δ*G* (kJ/mol)**				**ln*K***			
			283.15 K	293.15 K	303.15 K	313.15 K	283.15 K	293.15 K	303.15 K	313.15 K
Phosphate	23.546	98.493	−2.714	−3.378	−4.169	−4.270	1.875	2.125	2.530	2.806

**Table 4:** Cross-peak symbols for synchronous and asynchronous spectra and their multiplication results in IM@BC.

Wave Number (cm^−1^)	Functional Group	Wave Number (cm^−1^)														
		Synchronous Spectral Cross-Peak Symbols					Asynchronous Spectral Cross-Peak Symbols					Multiplication Results of Synchronous and Asynchronous Spectra				
		1525	1437	1150	1056	564	1525	1437	1150	1056	564	1525	1437	1150	1056	564
1525	-OH		+	-	-	-		+	-	+	+		+	+	-	-
1437	O=C-O			-	-	-			-	+	+			+	-	-
1150	C-O				-	-				+	+				-	-
1056	P-O					+					+					+
564	M-O															

## Data Availability

All the data have been included in the study.
